# The Impact of Kinases in Amyotrophic Lateral Sclerosis at the Neuromuscular Synapse: Insights into BDNF/TrkB and PKC Signaling

**DOI:** 10.3390/cells8121578

**Published:** 2019-12-05

**Authors:** Maria A. Lanuza, Laia Just-Borràs, Erica Hurtado, Víctor Cilleros-Mañé, Marta Tomàs, Neus Garcia, Josep Tomàs

**Affiliations:** Unitat d’Histologia i Neurobiologia (UHNEUROB), Universitat Rovira i Virgili, Facultat de Medicina i Ciències de la Salut, Sant Llorenç 21, 43201 Reus, Spain; laia.just@urv.cat (L.J.-B.); hc.erica@gmail.com (E.H.); victor.cilleros@urv.cat (V.C.-M.); marta.tomas@urv.cat (M.T.); mariadelesneus.garcia@urv.cat (N.G.)

**Keywords:** BDNF, TrkB, PKC, kinase, neuromuscular, NMJ, ALS, exercise

## Abstract

Brain-derived neurotrophic factor (BDNF) promotes neuron survival in adulthood in the central nervous system. In the peripheral nervous system, BDNF is a contraction-inducible protein that, through its binding to tropomyosin-related kinase B receptor (TrkB), contributes to the retrograde neuroprotective control done by muscles, which is necessary for motor neuron function. BDNF/TrkB triggers downstream presynaptic pathways, involving protein kinase C, essential for synaptic function and maintenance. Undeniably, this reciprocally regulated system exemplifies the tight communication between nerve terminals and myocytes to promote synaptic function and reveals a new view about the complementary and essential role of pre and postsynaptic interplay in keeping the synapse healthy and strong. This signaling at the neuromuscular junction (NMJ) could establish new intervention targets across neuromuscular diseases characterized by deficits in presynaptic activity and muscle contractility and by the interruption of the connection between nervous and muscular tissues, such as amyotrophic lateral sclerosis (ALS). Indeed, exercise and other therapies that modulate kinases are effective at delaying ALS progression, preserving NMJs and maintaining motor function to increase the life quality of patients. Altogether, we review synaptic activity modulation of the BDNF/TrkB/PKC signaling to sustain NMJ function, its and other kinases’ disturbances in ALS and physical and molecular mechanisms to delay disease progression.

## 1. Introduction

Neuromuscular disorders refer to different syndromes and diseases that impair skeletal muscle function. One of the most common neuromuscular disorders is amyotrophic lateral sclerosis (ALS), a neurodegenerative disease that progresses from a subtle decline in motor function to lethal respiratory paralysis within a few years of diagnosis [1]. Its onset is in adulthood, usually after the age of 50. It has a prevalence of about five people per 100,000 worldwide and is slightly more frequent in men. The disease can be familial (10%) and caused by dominant mutations in one of several genes, including *SOD1*, *C9orf72*, *TDP43* and *FUS* among others [1]. However, the disease is sporadic in most cases (90%).

Corticospinal tract loss, involving both upper and lower motor neuron (MN) death is a hallmark feature of ALS. Despite that nowadays it is impossible to define a chronological order of neuron affectation equally for all patients, the loss of neuromuscular junctions (NMJs)—the cholinergic synapses between lower MN and skeletal muscles—occurs before, and seems to be the primary cause of motor paralysis in both familial and sporadic forms of ALS [2,3,4,5]. The loss of a correct nerve-muscle contact significantly contributes to motor impairment and leads to pathological non-communication between the two tissues in different diseases. However, despite of data describing MN degeneration and early NMJ alterations through mechanisms such as axonal transport disruption, genomic and proteomic changes, abnormal cellular metabolism and tropism during ALS pathogenesis [6], few studies have addressed the changes occurring at the NMJ. Consequently, the pathological mechanisms leading to the detachment of motor nerve terminals from the muscle cells that result in NMJ degeneration are still poorly understood. Nevertheless, because NMJs are the first weakened points, any approach to preserve them can be valuable, especially if applied from the beginning of the disease.

The signaling pathways that control attachment and communication of motor axon terminals to muscle are only beginning to be understood, but neurotrophins, and especially, the brain-derived neurotrophic factor (BDNF)/tropomyosin-related kinase B receptor (TrkB) signaling and its downstream pathways, play important roles. This review focuses on BDNF/TrkB signaling at the neuromuscular system, its alteration in ALS and on therapeutic approaches designed to preserve NMJs maintain motor function to preserve lower MNs and increase the life quality of patients.

## 2. The NMJ Is Essential in Nerve–Muscle Bidirectional Communication

**Figure 1:**
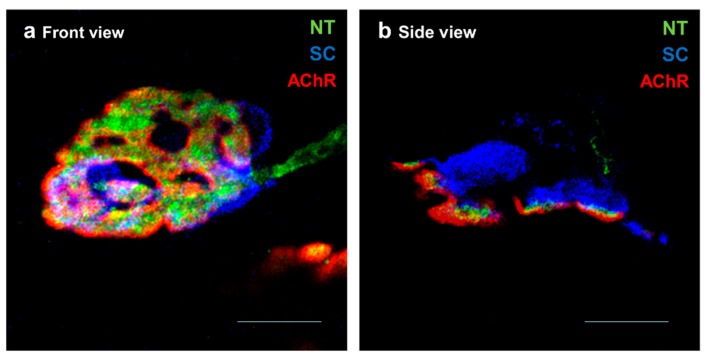
Cellular components of the neuromuscular junction (NMJ). Representative confocal micrographs of healthy NMJs from levator auris longus muscle showing a NMJ in a front view in (**a**) and an NMJ in side view in (**b**). The synapses are multiply immunofluorescent-stained: SNAP-25 in green to stain the nerve terminal (NT); S100 in blue to stain the Schwann cells (SC) and AChRs in red to stain the postsynaptic membrane. Scale bars = 10 μm.

The neuromuscular system is a complex and interconnected network that links the nervous system with skeletal musculature. It comprises individual motor units, each one integrating one α-motor neuron and all the myocytes it innervates [7]. Thus, motor unit elements are the indivisible quantal elements in all movements, as each action potential in the MN activates all the fibers. Furthermore, the interaction between the two tissues is fundamental for their health. On the one hand, presynaptic MNs and Schwann cells indicate to skeletal muscles how to grow, differentiate and function. On the other hand, skeletal muscles, which are essential for postural retention and locomotion, function as an endocrine organ to produce myokines that work both autocrinally and paracrinally over MNs, to sustain them during development, maintenance and disease and reinforce NMJs [8,9]. Indeed, recent research shows that signals from the skeletal muscles to the brain are as important as the signals from the brain to the muscles. This bidirectional communication between the two tissues starts at the NMJ and is fundamental for the health of its three cellular components (Figure 1). To control it, two fundamental mechanisms work coordinately to link the nervous system and skeletal muscles. They are the synaptic control, by which muscle contraction is initiated by nerve impulses generated in the central nervous system (CNS), and the neurotrophic control. We have recently shown that the two mechanisms regulate each other to modulate the synapse functionality and make it more efficient and stable.

Different signaling pathways control the structural connection between pre and postsynaptic components of the NMJ, contributing to its stability. One of the best characterized molecular mechanisms is the agrin/lipoprotein receptor-related protein 4 (Lrp4)/muscle-specific tyrosine kinase receptor (MuSK)/rapsyn pathway [10], which is essential for stabilizing acetylcholine receptors (AChR) clusters at synaptic sites [11,12]. Extracellular matrix proteins (such as laminins β2, α4 and α5; collagens IV and XIII) are also important for regulating the proper alignment between pre and postsynaptic membranes [13,14,15,16]. Neural cell adhesion molecules [17], ErbB kinase receptors [18,19] and many of the pathways required for NMJ formation [20,21] are also involved in its maintenance.

Multiple retrograde signals released by myofibers and Schwann cells that regulate presynaptic differentiation and maturation may play significant roles in NMJ adult stability. Among others, those include fibroblast growth factors, glial cell-derived neurotrophic factor (GDNF), transforming growth factor β (TGFβ) and neurotrophins [22,23,24,25,26]. The neurotrophic factor signaling at the neuromuscular synapses involve MN, muscle fibers and perisynaptic glia [27,28,29].

Among the neurotrophins, BDNF signaling is one of the most studied and representative of the neurotrophic control that regulates the development, differentiation, survival and function of neuromuscular system components [30]. Throughout the last few years, we have been studying several metabotropic signaling pathways inside the presynaptic terminal that could be involved in the control of NMJ bidirectional communication, especially regarding to those that converge on common intracellular kinases of the exocytotic machinery and that could be regulated by the BDNF signaling. This includes muscarinic acetylcholine autoreceptors (mAChRs) [31,32,33,34,35], TrkB [36] and adenosine receptors (AR) [37]. Moreover, we widely investigated the functional complex network constituted by these receptors and the presynaptic serine-threonine kinases, PKC and PKA, to regulate ACh release at the NMJ [38]. Furthermore, we presented the individual and complementary actions of presynaptic activity and the resulting muscle contraction over presynaptic kinases, and showed that BDNF/TrkB signaling is key in their regulation [36,39,40], which reveals the mutual intercommunication and regulation between the nerve terminal and the myocyte activities.

## 3. BDNF/TrkB Signaling in the NMJ Is Regulated by Pre and Postsynaptic Activity

**Figure 2:**
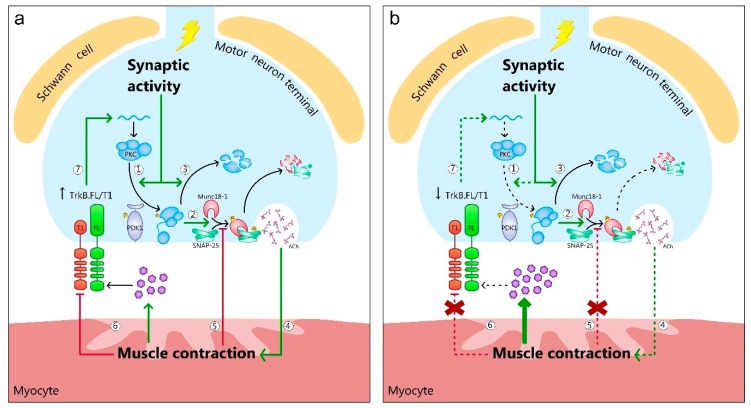
The BDNF/TrkB feedback signaling at the NMJs of healthy and amyotrophic lateral sclerotic (ALS) animals. (**a**) Healthy animals. Synaptic activity promotes PKC phosphorylation in the membrane through PDK1 (1). This results in the phosphorylation of PKC targets implicated in exocytosis, such as Munc18-1 and SNAP-25 (2). Once PKC has executed its action, it is typically degraded, decreasing its protein level (3). Consequently, ACh is released to reach its receptors in the junctional folds and trigger muscle contraction (4). Muscle contraction functions as a feedback loop to inhibit PKC phosphorylating activity over Munc18-1 and SNAP-25 (5). Moreover, it increases TrkB.FL signaling by secreting BDNF and decreasing TrkB.T1 levels and its dominant negative way to decrease TrkB.FL signaling (6). Therefore, an increased ratio TrkB.FL/T1 stimulates PKC synthesis in the presynaptic terminal, restoring its levels after activity-induced consumption (7). (**b**) ALS animals. Synaptic activity-induced PKC phosphorylation in the membrane through PDK1 is decreased (1). However, the levels of phosphorylated PKC targets implicated in exocytosis, such as Munc18-1 and SNAP-25 are increased (2), indicating that low PKC activity is functional. Moreover, PKC total levels are decreased, suggesting a minor synthesis or an increased degradation activity (3). Despite of the enhanced levels of phosphorylated exocytotic molecules, ACh release, and consequently, muscle contraction are decreased (4), suggesting a non-functional accumulation of these molecules. Therefore, muscle contraction loses the feedback loop to inhibit PKC’s phosphorylating activity (5), further contributing to the increase of Munc18-1 and SNAP-25 phosphorylated levels. Finally, despite of increased BDNF levels, maybe as a compensatory mechanism for TrkB misbalance, muscle contraction decreases the TrkB.FL signaling because the inhibiting action over TrkB.T1 is lost (6). Therefore, TrkB.FL’s effect on PKC synthesis is decreased, (7) contributing to the decrease of PKC levels.

BDNF/TrkB signaling is implicated in synaptic maintenance and neuronal survival in the CNS [41]. In the peripheral nervous system (PNS), TrkB has been implicated in the maintenance of the synaptic function and the structural integrity in adult muscular synapses [42]. Moreover, TrkB signaling also plays an important role in the neuromuscular bidirectional communication, as both the synaptic activity and the postsynaptic muscle contraction potentiate its action to activate presynaptic kinases on demand to control ACh release [36,39,40] (Figure 2a).

BDNF is strongly expressed in the brain [43], and, to a lesser extent, in skeletal muscle [27]. It is initially synthesized as a precursor protein (proBDNF) and subsequently cleaved into mature BDNF (mBDNF). Each BDNF isoform binds distinct receptors to mediate divergent neuronal actions [23,44,45,46]. ProBDNF preferentially interacts with p75^NTR^, whereas mBDNF selectively binds and activates its specific receptor TrkB. There are several isoforms of TrkB, generated by alternative splicing, with the same affinity to neurotrophins, including one full length isoform (TrkB.FL) and two truncated ones (TrkB.T1 and TrkB.T2), which lack part of the intracellular kinase domain [47,48]. Evidence suggests that heterodimers of TrkB.FL with the truncated isoforms inhibit trans-autophosphorylation of TrkB.FL, reducing BDNF signaling [49,50,51,52,53]. Despite some studies suggesting unique signaling roles for TrkB.T1, including modulating Ca^2+^ signaling in astrocytes [52], in the skeletal muscle, TrkB.T1 is the main isoform and acts in a dominant negative fashion to decrease TrkB.FL signaling [51,54,55]. Dorsey et al. (2012) [50] demonstrated that the deletion of TrkB.T1 increases neurotrophin-dependent activation of downstream signaling targets (e.g., Akt and p70/S6 kinase) and increases contractibility.

BDNF transcription, translation and secretion is strongly regulated by neural activity. Specifically, exercise training increases BDNF mRNA and protein expression in spinal cord and skeletal muscle in rodents [56,57,58,59,60,61] and basal neuromuscular activity is required to maintain normal levels of BDNF in the NMJ [58]. Furthermore, we showed that, in skeletal muscle, BDNF increases after presynaptic activity and even more after the resulting muscle contraction [36]. Accordingly, Mathews et al., 2009 [27] demonstrated that BDNF mRNA and protein expression is increased in electrically stimulated myotubes. Therefore, in vitro and in vivo experiments demonstrate that BDNF is a contraction-inducible protein. Moreover, Liem et al. (2001) [62] showed that mRNA is only located inside myocytes, contributing to the idea of a postsynaptic origin through BDNF mRNA translation that is directly linked to myofibril contraction.

There is no evidence showing that the BDNF produced by the skeletal muscle reaches the bloodstream. Therefore, it is not clear that is BDNF that directly induces a response in the brain [27,63]. Consequently, one can suggest that activity-induced BDNF synthesized by muscles acts paracrinally over nerve terminals by binding to TrkB receptors at the NMJ. Neuronal activity has been shown to rapidly activate and potentiate TrkB signaling, due to activity dependent secretion of BDNF [64,65,66]. Specifically, physical activity increases TrkB.FL mRNA levels in skeletal muscles and the spinal cord [58,60,67]. Moreover, we found that muscle contraction per se downregulates TrkB.T1 (decreasing the ratio to FL/T1) without changing TrkB.FL or p75^NTR^ levels. Therefore, it appears that the ratio between TrkB.FL and TrkB.T1 could determine the net effect of BDNF signaling at the neuromuscular system [36].

BDNF binds to TrkB.FL and activates its intrinsic tyrosine kinase domain, leading to autophosphorylation in the activation loop (tyr701, tyr706 and tyr707; [43,68]). The phosphorylation of these residues triggers the transphosphorylation of others tyrosine residues [69,70], tyr515 and tyr816 being the most extensively studied phosphorylation sites [71,72]. At the NMJ, synaptic activity induces a quick increase in pTrkB.FL at tyr816 while muscle contraction decreases it by increasing phosphatase activity [36]. These results suggest a complex mechanism regulating phosphorylation of TrkB.FL that involves complementary actions of the pre and postsynaptic activities.

One of the most important signaling pathways triggered by the phosphorylation of TrkB-FL in the tyr816 residue, after the BDNF binding, is the PLCγ one, which produces diacylglycerol (DAG) and inositol trisphosphate (IP3) [73,74]. IP3 leads to Ca^2+^ release, and, as a result, mature protein kinases C (PKCs) are cleaved to the membrane, leading to massive conformational changes in them, allowing for substrate binding, phosphorylation and downstream signaling effectors’ activations [75,76]. However, before PKCs can be functional, they need to be phosphorylated by PDK1 [77,78,79,80,81]. PDK1 activates multiple signaling pathways and seems to be constitutively active at the NMJ [82]. PDK1-induced PKC phosphorylation is enhanced by synaptic activity but is not directly linked to BDNF/TrkB signaling, which is, contrarily, involved in PKC activation once the isoforms are phosphorylated and mature [36].

PKC isoforms are differently regulated and localized in the NMJ [83]. Depending on the isoform, they require Ca^2+^ and/or diacylglycerol and phosphatidyl serine. Two PKC isoforms, nPKCε and cPKCβI, are exclusively presynaptic at the NMJ and participate in regulating ACh release [36,84,85,86]. Thus, they are ideal targets for both presynaptic and retrograde neurotrophic control induced by muscle contraction activity. Presynaptic activity decreases nPKCε and cPKCβI by an activation-induced degradation mechanism and muscle contraction increases its protein levels and both mechanisms are linked to the BDNF/TrkB activity [36,84,85,86]. This evidences the cross-linking between the pre and postsynaptic activities and the neurotrophic retrograde control to maintain a stable and sufficient pool of these PKC isoforms at the nerve terminal. Synaptic activity and BDNF/TrkB signaling directly increase the actions of nPKCε and cPKCβI, probably linked to the regulation of neurotransmission process, as the isoforms maintain and potentiate, respectively, the ACh release at the NMJ [36,86].

In skeletal muscles, nerve-induced stimulation, high Ca^2+^ concentrations and muscarinic signaling couple PKC to result in neurotransmitter release at the adult NMJ [84,87,88]. In particular, nPKCε maintains ACh release and modulates muscarinic signaling [86], while cPKCβI enhances neurotransmission [82] at the NMJ. Furthermore, evidence supports the importance of neurotrophic signaling via TrkB activity to regulate neuromuscular ACh release at short times and to maintain synaptic function and structural integrity in adult rodent NMJs long term [28,36,42,89,90]. Moreover, electrophysiological studies also demonstrated that the TrkB pathway needs an operative PKC to enhance ACh release, and PKA and PKC actions are related [91]. These effects of BDNF/TrkB signaling on neuromuscular transmission involve the interaction with presynaptic adenosine A2A receptors [92] and mAChRs [90,93]. We know that the normal function of the mAChR mechanism is a permissive prerequisite for the TrkB pathway to couple to ACh release. Reciprocally, the normal function of TrkB modulates M_1_ and M_2_-subtype muscarinic pathways [90]. However, the molecular interaction between TrkB and mAChRs to modulate ACh release in motor nerve terminals and how neuromuscular activity can modulate this relationship is still unknown.

A molecular approach recently demonstrated that several key proteins of the exocytotic mechanism of the synaptic vesicles at the NMJ are targets of nPKCε and cPKCβI [39,40]. In brief, the activity-induced phosphorylation of Munc18-1, a regulatory molecule of the exocytotic apparatus in the nerve terminals, depends on the coordinated action of both kinases and involves retrograde BDNF/TrkB signaling [39]. Moreover, SNAP-25, a member of the soluble N-ethyl maleimide sensitive-factor attachment protein receptors (SNARE) complex, is only phosphorylated by nPKCε independently of the neurotrophic control [40]. These pieces of evidence show that the activity-induced phosphorylation of molecules that are essential for neurotransmitter secretion at the NMJ depend on the synaptic activity-induced action of presynaptic PKCs that are controlled retrogradely by the BDNF/TrkB, which in turn is enhanced by the pre and postsynaptic activities. This accurately and reciprocally regulated molecular system is an example of the tight communication between nerve terminals and myocytes to promote synaptic function and opens a new view of the complementary and essential role of pre and postsynaptic interplay to contribute to keeping the synapse healthy and strong. Altogether, this signaling at the NMJ could establish new intervention targets across neuromuscular diseases characterized by deficits in presynaptic activity and muscle contractility and by the interruption of the connection between nervous and muscular tissues, such as ALS.

## 4. BDNF/TrkB Signaling Is Impaired in ALS NMJ

Due to the relevance of the BDNF/TrkB signaling pathway in synapses’ regulation and maintenance, especially regarding the NMJ, it has been studied in pathologies where synaptic function is impaired. Specifically, the BDNF/TrkB signaling is strongly impaired in brains affected by ALS, and all the approaches used around it were summarized in a recent review [94]. The present review summarizes how the retrograde BDNF/TrkB signaling pathway and the downstream alteration of protein kinases C is disturbed from its physiological mechanism in ALS at the NMJ and how exercise and other therapies that modulate kinases are effective at delaying ALS’s progression.

We recently showed the strong impairment of the BDNF/TrkB signaling at the plantaris muscle, one of the most affected by the disease [95] (Figure 2b). The molecular changes observed in the BDNF/TrkB pathway at the end stage of ALS may be either the cause or the consequence of the disease. However, it has key relevance, as in unaffected extraocular muscles in ALS mice it remains unaltered [96], while in limb muscles BDNF is accumulated because of TrkB.FL (and pTrkB.FL) reduction in favor of the negative TrkB.T1 isoform [95]. The resistance of extraocular muscles and MN results from their differences in relation to the rest of the musculature, including different myosin heavy chains and multi-innervated fibers even in mature individuals [97]. In symptomatic ALS animals, MN loss mainly affects the medium and large ones, innervating fast-twitching muscles in parallel with muscle phenotype changes [98], inducing a fast-to-slow transition from type II to type I fibers, and, within the type II population, from type IIb/IIx to IIa fibers [98,99]. Some of the alterations regarding BDNF and TrkB, but not all of them, respond to this fast-to-slow transition. They could be directly related with the etiology of ALS because among many possible causes, a genetic (or epigenetic, in sporadic ALS) change in the free-radical defense enzyme SOD1 could be detrimental for TrkB expression and turnover in MNs or NMJs and impair neuromuscular activity. However, some others may represent an adaptative effort to reduce the impairment of the neuromuscular function, as the levels of protein at the end stage of the disease describe the molecular pattern of survivor motor units.

ALS patients and mice have increased BDNF in skeletal muscle and the spinal cord [95,100,101]. This seems to be the result of the remaining slow muscle fibers that predominantly express BDNF [102] to function as a compensatory mechanism for the TrkB isoforms’ misbalance. Indeed, patients have decreased pTrkB.FL [100,101] despite increased total TrkB mRNA in the spinal cord [101]. However, functional TrkB.FL is lost in favour of TrkB.T1 increase [95]. Indeed, some of the changes are already found in presymptomatic mice [95], which may lead to an early impaired neuromuscular function [3,103] underlying forthcoming motoneuron loss. This suggestion is supported by experiments in which TrkB.T1 deletion in mutant SOD1 mice delays the onset of the disease, slows down the motoneuron loss and improves mobility tests results at the end stage of the disease compared with normal mutant SOD1 mice [104]. In addition, the deletion of TrkB.T1 increases neuromuscular function and nerve-evoked muscle contraction in healthy mice [50]. Altogether, the over-presence of TrkB.T1 limits the neurotrophic effect by masking TrkB.FL, with a direct impact on its autophosphorylation and signal transduction. Because of that, it seems that the increase of BDNF in ALS is an insufficient compensatory mechanism to promote neuronal survival of injured MNs.

Indeed, in normal mice, muscle-specific deletion of BDNF did not induce any change in NMJ integrity in healthy animals [105] nor did it have the same effect as TrkB disruption, which adversely affects NMJ structure and function [42,54,106,107]. This could decrease the importance of BDNF/TrkB signaling for maintaining NMJ function. However, this could be in accordance with results showing that exogenous BDNF and ALS-induced BDNF accumulation do not induce, by themselves, an improvement in patients nor mice [108,109]. Consequently, the activation of TrkB may be, rather than BDNF levels, the critical mechanism to influence the NMJ maintenance. Thus, this adds new evidence of the relevance of BDNF being able to work, which spotlights the importance of the mechanism of activation of TrkB, which depends on the TrkB.FL/TrkB.T1 ratio. Indeed, in the absence of BDNF, TrkB can be transactivated through A2R [110] and mAChR [90] and has been proposed as a therapy for neurodegenerative diseases [94].

Furthermore, Neurotrophin-4 (NT4) can also initiate intracellular signaling through TrkB [111]. It is significatively increased in ALS NMJ, probably because it is strongly expressed in slow, type I muscular fibers [102], which correlates with the fast-to-slow shift done by fast ALS affected fast muscles and with the surviving fibers. Furthermore, NT4 expression also depends on NMJ activity and increases in skeletal muscle electrically stimulated through motor nerves [102]. Thus, it could be possible that under BDNF lack, NT4 could activate TrkB instead as a compensation.

Moreover, p75^NTR^ regulates neuronal survival and death by binding to different ligands and co-receptors [112,113]. It promotes survival by binding Trk receptor and enhancing its ability to bind and respond to neurotrophins [113] and mediates cell death by binding to other receptors and proneurotrophins [114,115]. In ALS disease, when p75^NTR^ is decreased at the NMJ [95], it is not able to bind functional TrkB [116,117] which is also decreased [95], and under high doses of neurotrophins, p75^NTR^ acquires a proapoptotic role which goes through the activation of caspase 3 [118,119]. Consequently, p75^NTR^ seems implicated in motoneuron death in humans and in SOD1-G93A. Indeed, the extracellular domain of p75^NTR^ was found to be increased in urine samples from SOD1-G93A mice and ALS patients, being higher in rapidly progressing patients than in slowly progressing ones. Thus, this p75^NTR^ domain has been proposed as a candidate biomarker to detect ALS [120]. In accordance, therapeutic blocking or genetic depletion of p75^NTR^ in SOD1-G93A mice delayed onset and improved motor function and survival [119,121].

In summary, the neurotrophic signaling that under normal conditions guarantees the stability and functionality of the NMJ through synaptic activity [36] is highly and precociously affected in ALS. Thus, recovering the BDNF signaling is a good option for ALS therapy, due to its strong pro-survival effects through TrkB and p75^NTR^ in developing and injured MN [122,123,124]. However, because intrathecally administered exogenous BDNF does not improve motor function and survival in ALS patients [108] or autonomic nervous system function [109,125], it is essential to target the altered alternative splicing of the TrkB, as it is strongly related to the maintenance of NMJs done by the bidirectional synaptic and neurotrophic controls, as has already been pointed out.

## 5. BDNF/TrkB Downstream Protein Kinases and Targets Are Impaired in the ALS NMJ

TrkB.FL stimulates the PLCγ, which activates PKC [36,51,71,126]. Consequently, in ALS, where TrkB.FL is downregulated, PKC activation is affected, yet at the presymptomatic stage in some isoforms [95], suggesting a profound and complex molecular implication in the progression of the disease. Furthermore, other presynaptic metabotropic receptors related with PLCβ (such as adenosine receptor A_1_ and muscarinic receptor M_1_) may contribute to the selective modulation of different PKC isoforms in the muscle, as their activity is also related with TrkB and PKC [127,128,129].

In ALS limb muscles, the constitutive upstream kinase for PKCs, the PDK1 activity, is enhanced, resulting in the consumption of the phosphorylated PDK1 [95]. This has a direct and unequal impact in PKC isoforms’ phosphorylation and activity. Several PKC isoforms located at the presynaptic and postsynaptic components that regulate neurotransmission at the healthy NMJ [36,84,85,86,130,131] are differently affected in ALS muscles [95,132] (Figure 2b), probably due to their different mechanisms of activation. Firstly, the major activation of nPKCθ is linked with the inhibition of its target ClC-1 chloride channel [132] that results in morphological alterations of the neuromuscular presynaptic terminals, a high turnover rate of AChR and NMJ dismantlement in ALS mice [133]. On the other hand, the imbalance of cPKCβI and nPKCε may directly affect neurotransmission at the NMJ as it coincides with a significant increase in the phosphorylation of some of their targets, such as pMunc18-1 ser313 and pSNAP-25 ser187, indicating a dysregulation of the exocytotic synaptic machinery, as they are directly linked to it [95]. The increased levels of both molecules may be due to the remaining MNs generating and accumulating large amounts of pMunc18-1 and pSNAP-25 to maintain neurotransmission. At the same time, a decrease of PKCs in spinal cord MNs affected by ALS has been associated with a selective degeneration of the largest MNs [134]. At the presymptomatic phase of ALS, endplate potentials’ (EPPs’) amplitude and quantal content are increased, suggesting an abnormal upregulation in Ca^2+^ levels in the nerve terminals [135]. This coincides with molecular results that report a high phosphorylating efficacy on SNAP-25, indicating a good operation of vesicle release to support the high quantal content in presymptomatic stage. However, high Ca^2+^ concentrations have been related with apoptosis due to ion imbalance [136,137] and sustained calcium-dependent PKC activation [138]. Indeed, increased levels of adducin, another PKC target that participates in NMJ stabilization, have been found in ALS patients [139], which is another piece of evidence that elevations of Ca^2+^ lead to NMJ destabilization. Thus, as expected, large MN progressively die and EPPs’ amplitude and quantal release is reduced [140,141]. This phenomenon would explain selective MN loss and the fast-to-slow transition, because small and slow MN EPPs have smaller quantal content than big and fast MNs [142]; that would make them more resistant.

## 6. Exercise Reduces the BDNF/TrkB/PKC Signaling Impairment in ALS NMJ

Through the years, the benefits of physical activity on the nervous system health, including improvements and effectiveness in the NMJ function [143,144,145], morphology [146] and ACh released [147] have been consolidated. Physical exercise promotes cellular adaptations in the brain, spinal cord and skeletal muscles that could counteract the oxidative stress complication in ALS [148]. In skeletal muscle, training reduces oxidative stress following exercise [149], increases the mitochondrial capacity [150] and increases the expression of neurotrophic factors [57] that could prevent motoneuron degeneration, preserve muscle innervation and inhibit muscle atrophy [151,152,153].

Consequently, exercise has been proposed as a therapy for ALS [154,155]. Because the impairment of the NMJ is crucial in ALS onset and progression, strategies that structurally and functionally preserve it may improve the health of the neuromuscular cell partners. Indeed, in SOD1-G93A mice, maintaining neuromuscular activity extends motor units’ survival along ALS progression in partially denervated muscles [156], and, in ALS patients, moderate exercise ameliorates the disease symptoms, improves functionality and delays the progression [154,155,157,158,159,160]. However, results are still controversial as only modest increases in transgenic mice survival have been reported after moderate exercise [154,155,157,161,162,163] but not after intense exercise [164,165], probably due to differential effects depending on exercise characteristics. Thus, it is still necessary to find the ideal paradigm that could preserve NMJ to rescue the cellular components.

Physical training results in limited muscular atrophy in ALS mice, and especially, a swimming based protocol delayed disease onset and MN loss and improved motor performance [98]. One of the molecular mechanisms underlying these benefits at the NMJ is the BDNF/TrkB signaling pathway [95,166]. In Just-Borras et al., (2019b) [166] we show that running and swimming-based training protocols reduce or completely normalize the BDNF accumulation, respectively. Despite that exogenous BDNF therapy is not beneficial for ALS [124,167], BDNF and other neurotrophins are synthesized in response to myocyte activity to ameliorate NMJ activity. However, it is not useful because of TrkB.FL downregulation and TrkB.T1 upregulation [51,54,95]. Indeed, the adverse effect of TrkB.T1 on ALS mice has been extensively demonstrated, and it seems that its over-presence limits the BDNF action because of its negative effect over TrkB.FL that results in pTrkB.FL not transducing the intracellular signaling [50,104]. In contrast, in trained animals, the increased TrkB.FL can trigger the pathway that recovers the downstream signaling [166]. Indeed, the same effect is found in the WT diaphragm when muscle contraction is increased [36], which suggests a general effect of the increased activity.

Furthermore, in ALS NMJ, physical training normalizes the activity balance between cPKCβI and nPKCε over their target Munc18-1 [166], contributing to the recovery of the regulatory action of this molecule in synaptic vesicle exocytosis. However, pSNAP-25 ser187 is still upregulated despite the training, which may be interpreted as an adaptation to guarantee an optimal recruitment of ready releasable vesicles [168] that may help to maintain neurotransmission. However, we cannot discard that the dephosphorylation or degradation of pSNAP-25 may be impaired in ALS and that training is not capable of recovering this mechanism.

In summary, the reduction, prevention or even overcompensation of the ALS-induced damaging molecular changes in the BDNF/TrkB pathway and the downstream proteins at the NMJ by exercise training [166] adds new molecular evidence of the benefits of physical exercise to reduce the impact of the disease, as these molecular changes are contemporary with improvements in animals’ conditions, where MN loss is reduced. Altogether, despite the controversial opinions on physical exercise as therapy in ALS, it seems that it is always beneficial, but with precise exercise-dependent outcomes.

## 7. Other Kinases Involved in NMJ Health in ALS

Phosphorylation is probably the most widely used cellular signal in biological processes. Consequently, it is not surprising that several kinases have been related with multiple human diseases such as ALS. In ALS, apart from TrkB and PKC, several other kinases participate in the functionality of the PNS. Consequently, it is incomprehensible that any attempt focused on exclusively modulating one single protein may result in a radical improvement and in an effective delay of disease progression, due to the multifactorial nature of ALS.

Therefore, here we briefly summarize some of the kinases that have been more strongly related with the impairment of the NMJ function in ALS. Thus, knowing their mechanism of action will allow a more general strategy, involving more pathways that could have a more effective outcome.

### 7.1. TBK1 and Ripk

One of the most studied kinases in ALS is the serine-threonine kinase TANK-binding kinase 1 (TBK1) [169,170,171]. In normal conditions, it phosphorylates optineurin, which promotes autophagy when it is phosphorylated and binds to TBK1 [172,173,174,175,176,177,178]. However, due to TBK1 haploinsufficiency in many ALS cases, optineurin is less phosphorylated [174,179,180] which leads to reduced TBK1-optineurin complex and depleted pool of available phosphorylated optineurin to act as an autophagy promoter.

Furthermore, optineurin suppresses the signaling of the receptor-interacting protein kinase 1 (Ripk1); that potentiates, together with Ripk3 and the mixed lineage kinase-like protein (MLKL), the necroptosis pathway. Therefore, the loss of optineurin results in an increased functionality of the Ripk1/Ripk3/MLKL necroptotic pathway, leading to progressive demyelination and axonal degeneration in the CNS [181]. Furthermore, Ripk1 and Ripk3 mediated axonal pathology has been observed in SOD1-G93A mice and ALS patients spinal cords [181]. Thus, Ripk1 and Ripk3 play a critical role in mediating progressive axonal degeneration and inhibiting them has been proposed as a treatment for ALS as it preserves NMJ [182,183].

### 7.2. MuSK

Muscle-specific kinase (MuSK) is one of the many proteins that sustain the connection between nerves and muscles to maintain the neuromuscular function [184]. Yet during development, it stimulates the attachment of the nerve terminals to the myocytes. Moreover, it participates in the Agrin/Lrp4 signaling pathway [10], which is essential for stabilizing AChR clusters at synaptic sites [11,12]. Consequently, damage or mutations in MuSK result in neuromuscular diseases [185]. On the contrary, activating MuSK with an antibody treatment preserves NMJ innervation in ALS [186,187,188,189], although there are dissenting results between lack of MN survival and NMJ preservation and motor activity and life span enhancement in ALS mice that could be attributed to the differential methodologies used, and they manifest the need for further investigation to find an effective therapy for ALS.

### 7.3. ErbB

The ErbB receptors are a group of tyrosine kinases receptors involved in cell growth and survival in response to neuregulins. They are necessary for the correct formation of axons and modulate neurotransmitter receptor expression, playing a role also in the formation and plasticity of the NMJ [18,19]. Furthermore, ErbB2 and ErbB3 receptors interact with the glycoprotein CD44, whose function is to transfer extracellular signals for growth factors, such as insulin-like growth factor, hepatocyte growth factor and neuregulin, toward inside of the cells. CD44 interacts with neuregulin receptors in embryonic Schwann cells of peripheral nerves, thereby affecting growth and survival of these cells. Thus, their change of expression in ALS could be related with the pathology [190]. Moreover, mutations in ErbB4 have been described in autosomal dominant familial and sporadic forms of ALS [191] with concomitant frontotemporal dementia [192]. This supports a possible pathogenic role of ErbB4 in ALS [193].

### 7.4. PINK1

ALS is a disease extensively related with oxidative stress and mitochondrial damage [194,195]. When induced by increased reactive oxygen species, production leads to the accumulation of the PTEN-induced kinase 1 (PINK1) on the outer mitochondrial membrane (OMM). There, PINK1 phosphorylates and activates Parkin, which is a ubiquitin ligase, to ubiquitinate OMM proteins. Afterward, optineurin binds to ubiquitinated proteins to induce autophagy of the damaged mitochondria. The direct consequence is that when one of this proteins is mutated in ALS, mitophagy is disrupted and may directly contribute to neurodegeneration [196,197,198] directly affecting NMJ and MN due to the high density of mitochondria in the nerve terminals, where they support synaptic function [199].

### 7.5. PI3K and AKT

The Phosphoinositide 3-kinase (PI3K)/Protein kinase B (Akt) pathway regulates, among other processes, cell survival. In ALS, it is downregulated by the phosphatidylinositol-3,4,5-trisphosphate 3-phosphatase (PTEN), which is strongly altered [200]. As a result, Akt total levels and activity are decreased in human skeletal muscle at the end stage of the disease [201]. In accordance, restoring Akt activity resulted in a protective effect by delaying hindlimb muscles denervation [202], which is coincident with its upregulation in surviving MNs [203].

## 8. Conclusions

**Figure 3:**
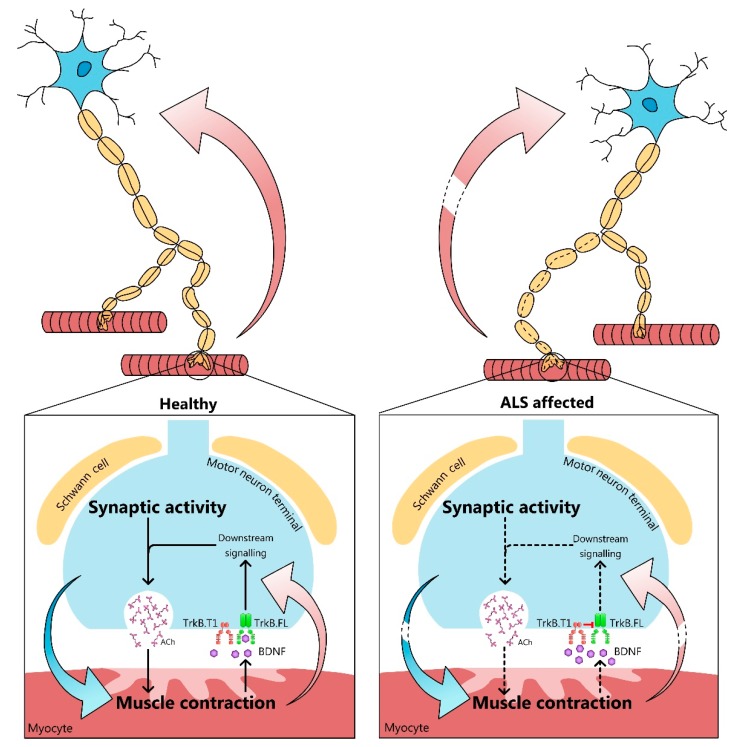
Concluding remarks. Pre and postsynaptic interplay is essential to preserving the NMJ to promote and sustain synaptic function through a tight communication between nerve terminals and myocytes. In physiological conditions (left), BDNF and TrkB trigger presynaptic pathways to modulate the synaptic function (pink arrows) that, meanwhile, stimulate muscle contraction (blue arrow). Consequently, a bidirectional collaboration is established to promote MNs and muscle function and survival. However, when the connection between nervous and muscular tissues is disrupted (right), as in ALS, deficits in presynaptic activity and muscle contractility appear. This results in NMJ dismantling, and, at the end stage of the disease, MN degeneration and skeletal muscle denervation and atrophy. Therefore, the absence of synaptic control induces molecular changes regarding neurotrophins, which lead to a loss of the neurotrophic control and, finally the deregulation of the neuromuscular system. Thus, pathologies that interrupt the communication between MN and myocytes highlight the importance of this double regulation. However, exercise and other future therapies based on recovering the affected molecular pathways are effective at delaying ALS progression, preserving NMJs and maintaining the bidirectional control between its elements (dashed lines in the arrows).

NMJ dismantling plays a crucial role in the onset of neuromuscular diseases like ALS, which is characterized by MN degeneration and death that ultimately leads to skeletal muscle denervation, atrophy, and, frequently, death of the patient within five years of diagnosis. Thus, apart from excitotoxicity and oxidative stress problems, ALS patients have to deal with a distal axonopathy, in which NMJ degeneration precedes and may even be related with MN loss. Several pieces of evidence supports that synaptic-specific mechanisms are partially responsible for the selective synaptic loss before MNs’ degeneration and recent research indicates that inherent characteristics of muscle fibers likely play an important part in the onset and progression of the disease. The BDNF/TrkB neurotrophic signaling is widely involved in the correct synapse maintenance and neuronal survival and strongly affected in ALS muscles. Consequently, the affection of BDNF/TrkB signaling in muscles with ALS disease deregulates presynaptic molecules—including PKCs and their targets—which could decrease synaptic activity, and therefore, the synaptic protection, and retrogradely affect MNs (Figure 2 and Figure 3). In this review, we propose that the loss of the tightly regulated nerve-muscle continuous communication in ALS pathology results in a functional motor impairment, muscle atrophy and paralysis. This corresponds with the increasing amount of biological evidence that during the last few years have revealed that the NMJ is a target for ALS therapies, which is a new vision of the disease. Furthermore, because ALS concerns several cellules from different structures, such as myocytes, lower and upper MNs, glia, etc., and has distinctive mechanisms and patterns of evolution, multifactorial personalized approaches are necessary.

## References

[1] Taylor J.P., Brown R.H., Cleveland D.W., Cleveland D.W. (2016). Decoding ALS: From genes to mechanism. Nature.

[2] Fischer L.R., Culver D.G., Tennant P., Davis A.A., Wang M., Castellano-Sanchez A., Khan J., Polak M.A., Glass J.D. (2004). Amyotrophic lateral sclerosis is a distal axonopathy: Evidence in mice and man. Exp. Neurol..

[3] Moloney E.B., de Winter F., Verhaagen J. (2014). ALS as a distal axonopathy: Molecular mechanisms affecting neuromuscular junction stability in the presymptomatic stages of the disease. Front. Neurosci..

[4] Pun S., Santos A.F., Saxena S., Xu L., Caroni P. (2006). Selective vulnerability and pruning of phasic motoneuron axons in motoneuron disease alleviated by CNTF. Nat. Neurosci..

[5] Schaefer A.M., Sanes J.R., Lichtman J.W. (2005). A compensatory subpopulation of motor neurons in a mouse model of amyotrophic lateral sclerosis. J. Comp. Neurol..

[6] Cleveland D.W., Williamson T.L. (1999). Slowing of axonal transport is a very early event in the toxicity ofALS–linked SOD1 mutants to motor neurons. Nat. Neurosci..

[7] Baldwin K.M., Haddad F., Pandorf C.E., Roy R.R., Edgerton V.R. (2013). Alterations in muscle mass and contractile phenotype in response to unloading models: Role of transcriptional/pretranslational mechanisms. Front. Physiol..

[8] Iizuka K., Machida T., Hirafuji M. (2014). Skeletal muscle is an endocrine organ. J. Pharm. Sci..

[9] Pedersen B.K., Febbraio M.A. (2012). Muscles, exercise and obesity: Skeletal muscle as a secretory organ. Nat. Rev. Endocrinol..

[10] Wu H., Xiong W.C., Mei L. (2010). To build a synapse: Signaling pathways in neuromuscular junction assembly. Development.

[11] Campanelli J.T., Hoch W., Rupp F., Kreiner T., Scheller R.H. (1991). Agrin mediates cell contact-induced acetylcholine receptor clustering. Cell.

[12] Gautam M., Noakes P.G., Moscoso L., Rupp F., Scheller R.H., Merlie J.P., Sanes J.R. (1996). Defective neuromuscular synaptogenesis in agrin-deficient mutant mice. Cell.

[13] Fox M.A., Sanes J.R., Borza D.-B., Eswarakumar V.P., Fässler R., Hudson B.G., John S.W.M., Ninomiya Y., Pedchenko V., Pfaff S.L. (2007). Distinct Target-Derived Signals Organize Formation, Maturation, and Maintenance of Motor Nerve Terminals. Cell.

[14] Nishimune H., Valdez G., Jarad G., Moulson C.L., Müller U., Miner J.H., Sanes J.R. (2008). Laminins promote postsynaptic maturation by an autocrine mechanism at the neuromuscular junction. J. Cell Biol..

[15] Nishimune H., Sanes J.R., Carlson S.S. (2004). A synaptic laminin–calcium channel interaction organizes active zones in motor nerve terminals. Nature.

[16] Singhal N., Martin P.T. (2011). Role of extracellular matrix proteins and their receptors in the development of the vertebrate neuromuscular junction. Dev. Neurobiol..

[17] Polo-Parada L., Bose C.M., Landmesser L.T. (2001). Alterations in Transmission, Vesicle Dynamics, and Transmitter Release Machinery at NCAM-Deficient Neuromuscular Junctions. Neuron.

[18] Lin W., Sanchez H.B., Deerinck T., Morris J.K., Ellisman M., Lee K.-F. (2000). Aberrant development of motor axons and neuromuscular synapses in erbB2-deficient mice. Proc. Natl. Acad. Sci. USA.

[19] Buonanno A., Fischbach G.D. (2001). Neuregulin and ErbB receptor signaling pathways in the nervous system. Curr. Opin. Neurobiol..

[20] Li L., Xiong W.-C., Mei L. (2018). Neuromuscular Junction Formation, Aging, and Disorders. Annu. Rev. Physiol..

[21] Liu W., Chakkalakal J.V. (2018). The Composition, Development, and Regeneration of Neuromuscular Junctions. Current Topics in Developmental Biology.

[22] Baudet C., Pozas E., Adameyko I., Andersson E., Ericson J., Ernfors P. (2008). Retrograde Signaling onto Ret during Motor Nerve Terminal Maturation. J. Neurosci..

[23] Je H.S., Yang F., Ji Y., Potluri S., Fu X.-Q., Luo Z.-G., Nagappan G., Chan J.P., Hempstead B., Son Y.-J. (2013). ProBDNF and mature BDNF as punishment and reward signals for synapse elimination at mouse neuromuscular junctions. J. Neurosci..

[24] Nguyen Q.T. (1998). Hyperinnervation of Neuromuscular Junctions Caused by GDNF Overexpression in Muscle. Science.

[25] Feng Z., Ko C.-P. (2008). Schwann cells promote synaptogenesis at the neuromuscular junction via transforming growth factor-beta1. J. Neurosci..

[26] Fuentes-Medel Y., Ashley J., Barria R., Maloney R., Freeman M., Budnik V. (2012). Integration of a Retrograde Signal during Synapse Formation by Glia-Secreted TGF-β Ligand. Curr. Biol..

[27] Matthews V.B., Åström M.-B., Chan M.H.S., Bruce C.R., Krabbe K.S., Prelovsek O., Åkerström T., Yfanti C., Broholm C., Mortensen O.H. (2009). Brain-derived neurotrophic factor is produced by skeletal muscle cells in response to contraction and enhances fat oxidation via activation of AMP-activated protein kinase. Diabetologia.

[28] Pitts E.V., Potluri S., Hess D.M., Balice-Gordon R.J. (2006). Neurotrophin and Trk-mediated signaling in the neuromuscular system. Int. Anesth. Clin..

[29] Funakoshi H. (1993). Differential expression of mRNAs for neurotrophins and their receptors after axotomy of the sciatic nerve. J. Cell Biol..

[30] Cisterna B.A., Cardozo C., Saez J.C. (2014). Neuronal involvement in muscular atrophy. Front. Cell. Neurosci..

[31] Minic J., Molgó J., Karlsson E., Krejci E. (2002). Regulation of acetylcholine release by muscarinic receptors at the mouse neuromuscular junction depends on the activity of acetylcholinesterase. Eur. J. Neurosci..

[32] Santafé M.M., Salon I., Garcia N., Lanuza M.A., Uchitel O.D., Tomàs J. (2003). Modulation of ACh release by presynaptic muscarinic autoreceptors in the neuromuscular junction of the newborn and adult rat. Eur. J. Neurosci..

[33] Santafé M.M., Salon I., Garcia N., Lanuza M.A., Uchitel O.D., Tomàs J. (2004). Muscarinic autoreceptors related with calcium channels in the strong and weak inputs at polyinnervated developing rat neuromuscular junctions. Neuroscience.

[34] Santafé M.M., Lanuza M.A., Garcia N., Tomàs J. (2005). Calcium inflow-dependent protein kinase C activity is involved in the modulation of transmitter release in the neuromuscular junction of the adult rat. Synapse.

[35] Slutsky I., Parnas H., Parnas I. (1999). Presynaptic effects of muscarine on ACh release at the frog neuromuscular junction. J. Physiol..

[36] Hurtado E., Cilleros V., Nadal L., Simó A., Obis T., Garcia N., Santafé M.M., Tomàs M., Halievski K., Jordan C.L. (2017). Muscle Contraction Regulates BDNF/TrkB Signaling to Modulate Synaptic Function through Presynaptic cPKCα and cPKCβI. Front. Mol. Neurosci..

[37] Song W.-J., Tkatch T., Surmeier D.J. (2000). Adenosine Receptor Expression and Modulation of Ca^2+^ Channels in Rat Striatal Cholinergic Interneurons. J. Neurophysiol..

[38] Tomàs J., Garcia N., Lanuza M.A., Santafé M.M., Tomàs M., Nadal L., Hurtado E., Simó-Ollé A., Cilleros-Mañé V., Just-Borràs L. (2018). Adenosine Receptors in Developing and Adult Mouse Neuromuscular Junctions and Functional Links With Other Metabotropic Receptor Pathways. Front. Pharm..

[39] Simó A., Just-Borràs L., Cilleros-Mañé V., Hurtado E., Nadal L., Tomàs M., Garcia N., Lanuza M.A., Tomàs J. (2018). BDNF-TrkB Signaling Coupled to nPKCε and cPKCβI Modulate the Phosphorylation of the Exocytotic Protein Munc18-1 During Synaptic Activity at the Neuromuscular Junction. Front. Mol. Neurosci..

[40] Simó A., Cilleros-Mañé V., Just-Borràs L., Hurtado E., Nadal L., Tomàs M., Garcia N., Lanuza M.A., Tomàs J. (2019). nPKCε Mediates SNAP-25 Phosphorylation of Ser-187 in Basal Conditions and After Synaptic Activity at the Neuromuscular Junction. Mol. Neurobiol..

[41] Snider W.D. (1998). How do you feel? Neurotrophins and mechanotransduction. Nat. Neurosci..

[42] Mantilla C.B., Stowe J.M., Sieck D.C., Ermilov L.G., Greising S.M., Zhang C., Shokat K.M., Sieck G.C. (2014). TrkB kinase activity maintains synaptic function and structural integrity at adult neuromuscular junctions. J. Appl. Physiol..

[43] Huang E.J., Reichardt L.F. (2003). Trk Receptors: Roles in Neuronal Signal Transduction. Annu. Rev. Biochem..

[44] Lu B. (2003). BDNF and Activity-Dependent Synaptic Modulation. Learn. Mem..

[45] Yang F., Je H.-S., Ji Y., Nagappan G., Hempstead B., Lu B. (2009). Pro-BDNF-induced synaptic depression and retraction at developing neuromuscular synapses. J. Cell Biol..

[46] Hempstead B.L. (2006). Dissecting the diverse actions of pro- and mature neurotrophins. Curr. Alzheimer Res..

[47] Reichardt L.F. (2006). Neurotrophin-regulated signalling pathways. Philos. Trans. R. Soc. B.

[48] Middlemas D.S., Lindberg R.A., Hunter T. (1991). trkB, a neural receptor protein-tyrosine kinase: Evidence for a full-length and two truncated receptors. Mol. Cell. Biol..

[49] Baxter G.T., Radeke M.J., Kuo R.C., Makrides V., Hinkle B., Hoang R., Medina-Selby A., Coit D., Valenzuela P., Feinstein S.C. (1997). Signal transduction mediated by the truncated trkB receptor isoforms, trkB.T1 and trkB.T2. J. Neurosci..

[50] Dorsey S.G., Lovering R.M., Renn C.L., Leitch C.C., Liu X., Tallon L.J., Sadzewicz L.D., Pratap A., Ott S., Sengamalay N. (2012). Genetic deletion of trkB.T1 increases neuromuscular function. Am. J. Physiol..

[51] Eide F.F., Vining E.R., Eide B.L., Zang K., Wang X.Y., Reichardt L.F. (1996). Naturally occurring truncated trkB receptors have dominant inhibitory effects on brain-derived neurotrophic factor signaling. J. Neurosci..

[52] Rose C.R., Blum R., Pichler B., Lepier A., Kafitz K.W., Konnerth A. (2003). Truncated TrkB-T1 mediates neurotrophin-evoked calcium signalling in glia cells. Nature.

[53] Wong J., Garner B. (2012). Evidence that truncated TrkB isoform, TrkB-Shc can regulate phosphorylated TrkB protein levels. Biochem. Biophys. Res. Commun..

[54] Gonzalez M., Ruggiero F.P., Chang Q., Shi Y.J., Rich M.M., Kraner S., Balice-Gordon R.J. (1999). Disruption of Trkb-mediated signaling induces disassembly of postsynaptic receptor clusters at neuromuscular junctions. Neuron.

[55] Haapasalo A., Koponen E., Hoppe E., Wong G., Castrén E. (2001). Truncated trkB.T1 Is Dominant Negative Inhibitor of trkB.TK+-Mediated Cell Survival. Biochem. Biophys. Res. Commun..

[56] Cuppini R., Sartini S., Agostini D., Guescini M., Ambrogini P., Betti M., Bertini L., Vallasciani M., Stocchi V. (2007). Bdnf expression in rat skeletal muscle after acute or repeated exercise. Arch. Ital. Biol..

[57] Gómez-Pinilla F., Ying Z., Opazo P., Roy R.R., Edgerton V.R. (2001). Differential regulation by exercise of BDNF and NT-3 in rat spinal cord and skeletal muscle. Eur. J. Neurosci..

[58] Gómez-Pinilla F., Ying Z., Roy R.R., Molteni R., Edgerton V.R. (2002). Voluntary Exercise Induces a BDNF-Mediated Mechanism That Promotes Neuroplasticity. J. Neurophysiol..

[59] Gomez-Pinilla F., Ying Z., Zhuang Y. (2012). Brain and Spinal Cord Interaction: Protective Effects of Exercise Prior to Spinal Cord Injury. PLoS ONE.

[60] Zoladz J.A., Pilc A. (2010). The effect of physical activity on the brain derived neurotrophic factor: From animal to human studies. J. Physiol. Pharm..

[61] Adlard P.A., Perreau V.M., Engesser-Cesar C., Cotman C.W. (2004). The timecourse of induction of brain-derived neurotrophic factor mRNA and protein in the rat hippocampus following voluntary exercise. Neurosci. Lett..

[62] Liem R., Brouwer N., Copray J. (2001). Ultrastructural localisation of intramuscular expression of BDNF mRNA by silver-gold intensified non-radioactive in situ hybridisation. Histochem. Cell Biol..

[63] Pedersen B.K. (2019). Physical activity and muscle–brain crosstalk. Nat. Rev. Endocrinol..

[64] Meyer-Franke A., Wilkinson G.A., Kruttgen A., Hu M., Munro E., Hanson M.G., Reichardt L.F., Barres B.A. (1998). Depolarization and cAMP elevation rapidly recruit TrkB to the plasma membrane of CNS neurons. Neuron.

[65] Aloyz R., Fawcett J.P., Kaplan D.R., Murphy R.A., Miller F.D. (1999). Activity-dependent activation of TrkB neurotrophin receptors in the adult CNS. Learn. Mem..

[66] Patterson S.L., Pittenger C., Morozov A., Martin K.C., Scanlin H., Drake C., Kandel E.R. (2001). Some forms of cAMP-mediated long-lasting potentiation are associated with release of BDNF and nuclear translocation of phospho-MAP kinase. Neuron.

[67] Skup M., Dwornik A., Macias M., Sulejczak D., Wiater M., Czarkowska-Bauch J. (2002). Long-term locomotor training up-regulates TrkB(FL) receptor-like proteins, brain-derived neurotrophic factor, and neurotrophin 4 with different topographies of expression in oligodendroglia and neurons in the spinal cord. Exp. Neurol..

[68] Guiton M., Gunn-Moore F.J., Stitt T.N., Yancopoulos G.D., Tavaré J.M. (1994). Identification of in vivo brain-derived neurotrophic factor-stimulated autophosphorylation sites on the TrkB receptor tyrosine kinase by site-directed mutagenesis. J. Biol. Chem..

[69] Cunningham M.E., Stephens R.M., Kaplan D.R., Greene L.A. (1997). Autophosphorylation of activation loop tyrosines regulates signaling by the TRK nerve growth factor receptor. J. Biol. Chem..

[70] Friedman W.J., Greene L.A. (1999). Neurotrophin Signaling via Trks and p75. Exp. Cell Res..

[71] Middlemas D.S., Meisenhelder J., Hunter T. (1994). Identification of TrkB autophosphorylation sites and evidence that phospholipase C-gamma1 is a substrate of the TrkB receptor. J. Biol. Chem..

[72] Segal R.A., Bhattacharyya A., Rua L.A., Alberta J.A., Stephens R.M., Kaplan D.R., Stiles C.D. (1996). Differential utilization of Trk autophosphorylation sites. J. Biol. Chem..

[73] Carpenter G., Ji Q. (1999). Phospholipase C-γ as a Signal-Transducing Element. Exp. Cell Res..

[74] Kleiman R.J., Tian N., Krizaj D., Hwang T.N., Copenhagen D.R., Reichardt L.F. (2000). BDNF-Induced potentiation of spontaneous twitching in innervated myocytes requires calcium release from intracellular stores. J. Neurophysiol..

[75] Colón-González F., Kazanietz M.G. (2006). C1 domains exposed: From diacylglycerol binding to protein-protein interactions. Biochim. Biophys. Acta.

[76] Griner E.M., Kazanietz M.G. (2007). Protein kinase C and other diacylglycerol effectors in cancer. Nat. Rev. Cancer.

[77] Balendran A., Hare G.R., Kieloch A., Williams M.R., Alessi D.R. (2000). Further evidence that 3-phosphoinositide-dependent protein kinase-1 (PDK1) is required for the stability and phosphorylation of protein kinase C (PKC) isoforms. FEBS Lett..

[78] Chou M.M., Hou W., Johnson J., Graham L.K., Lee M.H., Chen C.-S., Newton A.C., Schaffhausen B.S., Toker A. (1998). Regulation of protein kinase C ζ by PI 3-kinase and PDK-1. Curr. Biol..

[79] Keranen L.M., Dutil E.M., Newton A.C. (1995). Protein kinase C is regulated in vivo by three functionally distinct phosphorylations. Curr. Biol..

[80] Le Good J.A., Ziegler W.H., Parekh D.B., Alessi D.R., Cohen P., Parker P.J. (1998). Protein kinase C isotypes controlled by phosphoinositide 3-kinase through the protein kinase PDK1. Science.

[81] Sonnenburg E.D., Gao T., Newton A.C. (2001). The phosphoinositide-dependent kinase, PDK-1, phosphorylates conventional protein kinase C isozymes by a mechanism that is independent of phosphoinositide 3-kinase. J. Biol. Chem..

[82] Hurtado E., Cilleros V., Just L., Simó A., Nadal L., Tomàs M., Garcia N., Lanuza M.A., Tomàs J. (2017). Synaptic Activity and Muscle Contraction Increases PDK1 and PKCβI Phosphorylation in the Presynaptic Membrane of the Neuromuscular Junction. Front. Mol. Neurosci..

[83] Lanuza M.A., Santafe M.M., Garcia N., Besalduch N., Tomàs M., Obis T., Priego M., Nelson P.G., Tomàs J. (2014). Protein kinase C isoforms at the neuromuscular junction: Localization and specific roles in neurotransmission and development. J. Anat..

[84] Besalduch N., Tomàs M., Santafé M.M., Garcia N., Tomàs J., Lanuza M.A. (2010). Synaptic activity-related classical protein kinase C isoform localization in the adult rat neuromuscular synapse. J. Comp. Neurol..

[85] Obis T., Besalduch N., Hurtado E., Nadal L., Santafe M.M., Garcia N., Tomàs M., Priego M., Lanuza M.A., Tomàs J. (2015). The novel protein kinase C epsilon isoform at the adult neuromuscular synapse: Location, regulation by synaptic activity-dependent muscle contraction through TrkB signaling and coupling to ACh release. Mol. Brain.

[86] Obis T., Hurtado E., Nadal L., Tomàs M., Priego M., Simon A., Garcia N., Santafe M.M., Lanuza M.A., Tomàs J. (2015). The novel protein kinase C epsilon isoform modulates acetylcholine release in the rat neuromuscular junction. Mol. Brain.

[87] Santafé M.M., Lanuza M.A., Garcia N., Tomàs J. (2006). Muscarinic autoreceptors modulate transmitter release through protein kinase C and protein kinase A in the rat motor nerve terminal. Eur. J. Neurosci..

[88] Santafé M.M., Lanuza M.A., Garcia N., Tomàs M., Tomàs J.M. (2007). Coupling of presynaptic muscarinic autoreceptors to serine kinases in low and high release conditions on the rat motor nerve terminal. Neuroscience.

[89] Mantilla C.B., Zhan W.-Z., Sieck G.C. (2004). Neurotrophins improve neuromuscular transmission in the adult rat diaphragm. Muscle Nerve.

[90] Garcia N., Tomàs M., Santafé M.M., Besalduch N., Lanuza M.A., Tomàs J. (2010). The interaction between tropomyosin-related kinase B receptors and presynaptic muscarinic receptors modulates transmitter release in adult rodent motor nerve terminals. J. Neurosci..

[91] Santafé M.M., Garcia N., Tomàs M., Obis T., Lanuza M.A., Besalduch N., Tomàs J. (2014). The interaction between tropomyosin-related kinase B receptors and serine kinases modulates acetylcholine release in adult neuromuscular junctions. Neurosci. Lett..

[92] Pousinha P.A., Diogenes M.J., Ribeiro J.A., Sebastião A.M. (2006). Triggering of BDNF facilitatory action on neuromuscular transmission by adenosine A2A receptors. Neurosci. Lett..

[93] Tomàs J., Santafé M.M., Garcia N., Lanuza M.A., Tomàs M., Besalduch N., Obis T., Priego M., Hurtado E. (2014). Presynaptic membrane receptors in acetylcholine release modulation in the neuromuscular synapse. J. Neurosci. Res..

[94] Pradhan J., Noakes P.G., Bellingham M.C. (2019). The Role of Altered BDNF/TrkB Signaling in Amyotrophic Lateral Sclerosis. Front. Cell. Neurosci..

[95] Just-Borràs L., Hurtado E., Cilleros-Mañé V., Biondi O., Charbonnier F., Tomàs M., Garcia N., Lanuza M.A., Tomàs J. (2019). Overview of Impaired BDNF Signaling, Their Coupled Downstream Serine-Threonine Kinases and SNARE/SM Complex in the Neuromuscular Junction of the Amyotrophic Lateral Sclerosis Model SOD1-G93A Mice. Mol. Neurobiol..

[96] Harandi V.M., Gaied A.R.N., Brännström T., Pedrosa Domellöf F., Liu J.-X. (2016). Unchanged Neurotrophic Factors and Their Receptors Correlate With Sparing in Extraocular Muscles in Amyotrophic Lateral Sclerosis. Investig. Opthalmology Vis. Sci..

[97] Nijssen J., Aguila J., Hoogstraaten R., Kee N., Hedlund E. (2018). Axon-Seq Decodes the Motor Axon Transcriptome and Its Modulation in Response to ALS. Stem Cell Rep..

[98] Deforges S., Branchu J., Biondi O., Grondard C., Pariset C., Lécolle S., Lopes P., Vidal P.-P., Chanoine C., Charbonnier F. (2009). Motoneuron survival is promoted by specific exercise in a mouse model of amyotrophic lateral sclerosis. J. Physiol..

[99] Hegedus J., Putman C.T., Tyreman N., Gordon T. (2008). Preferential motor unit loss in the SOD1 G93A transgenic mouse model of amyotrophic lateral sclerosis. J. Physiol..

[100] Küst B.M., Copray J.C.V.M., Brouwer N., Troost D., Boddeke H.W.G.M. (2002). Elevated Levels of Neurotrophins in Human Biceps Brachii Tissue of Amyotrophic Lateral Sclerosis. Exp. Neurol..

[101] Mutoh T., Sobue G., Hamano T., Kuriyama M., Hirayama M., Yamamoto M., Mitsuma T. (2000). Decreased Phosphorylation Levels of TrkB Neurotrophin Receptor in the Spinal Cords from Patients with Amyotrophic Lateral Sclerosis. Neurochem. Res..

[102] Funakoshi H., Belluardo N., Arenas E., Yamamoto Y., Casabona A., Persson H., Ibanez C. (1995). Muscle-derived neurotrophin-4 as an activity-dependent trophic signal for adult motor neurons. Science.

[103] Campanari M.-L., García-Ayllón M.-S., Ciura S., Sáez-Valero J., Kabashi E. (2016). Neuromuscular Junction Impairment in Amyotrophic Lateral Sclerosis: Reassessing the Role of Acetylcholinesterase. Front. Mol. Neurosci..

[104] Yanpallewar S.U., Barrick C.A., Buckley H., Becker J., Tessarollo L. (2012). Deletion of the BDNF Truncated Receptor TrkB.T1 Delays Disease Onset in a Mouse Model of Amyotrophic Lateral Sclerosis. PLoS ONE.

[105] Delezie J., Weihrauch M., Maier G., Tejero R., Ham D.J., Gill J.F., Karrer-Cardel B., Rüegg M.A., Tabares L., Handschin C. (2019). BDNF is a mediator of glycolytic fiber-type specification in mouse skeletal muscle. Proc. Natl. Acad. Sci. USA.

[106] Chevrel G., Hohlfeld R., Sendtner M. (2006). The role of neurotrophins in muscle under physiological and pathological conditions. Muscle Nerve..

[107] Kulakowski S.A., Parker S.D., Personius K.E. (2011). Reduced TrkB expression results in precocious age-like changes in neuromuscular structure, neurotransmission, and muscle function. J. Appl. Physiol..

[108] Ochs G., Penn R.D., York M., Giess R., Beck M., Tonn J., Haigh J., Malta E., Traub M., Sendtner M. (2000). A phase I/II trial of recombinant methionyl human brain derived neurotrophic factor administered by intrathecal infusion to patients with amyotrophic lateral sclerosis. Amyotroph. Lateral Scler. Other Mot. Neuron Disord..

[109] Beck M., Flachenecker P., Magnus T., Giess R., Reiners K., Toyka K.V., Naumann M. (2005). Autonomic dysfunction in ALS: A preliminary study on the effects of intrathecal BDNF. Amyotroph. Lateral Scler. Other Mot. Neuron Disord..

[110] Lee F.S., Chao M. (2001). V Activation of Trk neurotrophin receptors in the absence of neurotrophins. Proc. Natl. Acad. Sci. USA.

[111] Klein R., Nanduri V., Jing S., Lamballe F., Tapley P., Bryant S., Cordon-Cardo C., Jones K.R., Reichardt L.F., Barbacid M. (1991). The trkB tyrosine protein kinase is a receptor for brain-derived neurotrophic factor and neurotrophin-3. Cell.

[112] Barker P.A. (2004). p75NTR is positively promiscuous: Novel partners and new insights. Neuron.

[113] Bibel M., Hoppe E., Barde Y.A. (1999). Biochemical and functional interactions between the neurotrophin receptors trk and p75NTR. Embo. J..

[114] Nykjaer A., Willnow T.E., Petersen C.M. (2005). p75NTR—live or let die. Curr. Opin. Neurobiol..

[115] Hu Y., Lee X., Shao Z., Apicco D., Huang G., Gong B.J., Pepinsky R.B., Mi S. (2013). A DR6/p75(NTR) complex is responsible for β-amyloid-induced cortical neuron death. Cell Death Dis..

[116] Lowry K.S., Murray S.S., McLean C.A., Talman P., Mathers S., Lopes E.C., Cheema S.S. (2001). A potential role for the p75 low-affinity neurotrophin receptor in spinal motor neuron degeneration in murine and human amyotrophic lateral sclerosis. Amyotroph. Lateral Scler. Other Mot. Neuron Disord..

[117] Dupuis L., Pehar M., Cassina P., Rene F., Castellanos R., Rouaux C., Gandelman M., Dimou L., Schwab M.E., Loeffler J.P. (2008). Nogo receptor antagonizes p75NTR-dependent motor neuron death. Proc. Natl. Acad. Sci. USA.

[118] Hempstead B.L. (2002). The many faces of p75NTR. Curr. Opin. Neurobiol..

[119] Turner B.J., Cheah I.K., Macfarlane K.J., Lopes E.C., Petratos S., Langford S.J., Cheema S.S. (2003). Antisense peptide nucleic acid-mediated knockdown of the p75 neurotrophin receptor delays motor neuron disease in mutant SOD1 transgenic mice. J. Neurochem..

[120] Shepheard S.R., Chataway T., Schultz D.W., Rush R.A., Rogers M.-L. (2014). The extracellular domain of neurotrophin receptor p75 as a candidate biomarker for amyotrophic lateral sclerosis. PLoS ONE.

[121] Küst B.M., Brouwer N., Mantingh I.J., Boddeke H.W.G.M., Copray J.C.V.M. (2003). Reduced p75NTR expression delays disease onset only in female mice of a transgenic model of familial amyotrophic lateral sclerosis. Amyotroph. Lateral Scler. Other Mot. Neuron Disord..

[122] Peng H.B., Yang J.-F., Dai Z., Lee C.W., Hung H.W., Feng Z.H., Ko C.-P. (2003). Differential effects of neurotrophins and schwann cell-derived signals on neuronal survival/growth and synaptogenesis. J. Neurosci..

[123] Mantilla C.B., Gransee H.M., Zhan W.-Z., Sieck G.C. (2013). Motoneuron BDNF/TrkB signaling enhances functional recovery after cervical spinal cord injury. Exp. Neurol..

[124] Nagahara A.H., Tuszynski M.H. (2011). Potential therapeutic uses of BDNF in neurological and psychiatric disorders. Nat. Rev. Drug Discov..

[125] Nichols N.L., Satriotomo I., Allen L.L., Grebe A.M., Mitchell G.S. (2017). Mechanisms of enhanced phrenic long-term facilitation in SOD1 G93a rats. J. Neurosci..

[126] Patapoutian A., Reichardt L.F. (2001). Trk receptors: Mediators of neurotrophin action. Curr. Opin. Neurobiol..

[127] Nadal L., Garcia N., Hurtado E., Simó A., Tomàs M., Lanuza M.A. (2017). Presynaptic Muscarinic Acetylcholine Receptors and TrkB Receptor Cooperate in the Elimination of Redundant Motor Nerve Terminals during Development. Front. Aging Neurosci..

[128] Garcia N., Priego M., Obis T., Santafe M.M., Tomàs M., Besalduch N., Lanuza Ma., Tomàs J. (2013). Adenosine A1 and A2A receptor-mediated modulation of acetylcholine release in the mice neuromuscular junction. Eur. J. Neurosci..

[129] Tomàs J., Garcia N., Lanuza M.A., Santafé M.M., Tomàs M., Nadal L., Hurtado E., Simó A., Cilleros V. (2017). Presynaptic Membrane Receptors Modulate ACh Release, Axonal Competition and Synapse Elimination during Neuromuscular Junction Development. Front. Mol. Neurosci..

[130] Li M.-X., Jia M., Yang L.-X., Jiang H., Lanuza M.A., Gonzalez C.M., Nelson P.G. (2004). The Role of the Theta Isoform of Protein Kinase C (PKC) in Activity-Dependent Synapse Elimination: Evidence from the PKC Theta Knock-Out Mouse In Vivo and In Vitro. J. Neurosci..

[131] Lanuza M.A., Besalduch N., González C., Santafé M.M., Garcia N., Tomàs M., Nelson P.G., Tomàs J. (2010). Decreased phosphorylation of δ and ε subunits of the acetylcholine receptor coincides with delayed postsynaptic maturation in PKC θ deficient mouse. Exp. Neurol..

[132] Camerino G.M., Fonzino A., Conte E., De Bellis M., Mele A., Liantonio A., Tricarico D., Tarantino N., Dobrowolny G., Musarò A. (2019). Elucidating the Contribution of Skeletal Muscle Ion Channels to Amyotrophic Lateral Sclerosis in search of new therapeutic options. Sci. Rep..

[133] Dobrowolny G., Martini M., Scicchitano B.M., Romanello V., Boncompagni S., Nicoletti C., Pietrangelo L., De Panfilis S., Catizone A., Bouchè M. (2018). Muscle Expression of SOD1 G93A Triggers the Dismantlement of Neuromuscular Junction via PKC-Theta. Antioxid. Redox Signal.

[134] Nagao M., Kato S., Oda M., Hirai S. (1998). Decrease of protein kinase C in the spinal motor neurons of amyotrophic lateral sclerosis. Acta. Neuropathol..

[135] Plomp J.J., Vergouwe M.N., Van den Maagdenberg A.M., Ferrari M.D., Frants R.R., Molenaar P.C. (2000). Abnormal transmitter release at neuromuscular junctions of mice carrying the tottering alpha1A Ca2+ channel mutation. Brain.

[136] Felipo V., Miñana M.D., Grisolía S. (1993). Inhibitors of protein kinase C prevent the toxicity of glutamate in primary neuronal cultures. Brain Res..

[137] Krieger C., Lanius R.A., Pelech S.L., Shaw C. (1996). a Amyotrophic lateral sclerosis: The involvement of intracellular Ca2+ and protein kinase C. Trends Pharm. Sci.

[138] Mondola P., Damiano S., Sasso A., Santillo M. (2016). The Cu, Zn Superoxide Dismutase: Not Only a Dismutase Enzyme. Front. Physiol..

[139] Krieger C., Hu J.H., Pelech S. (2003). Aberrant protein kinases and phosphoproteins in amyotrophic lateral sclerosis. Trends Pharm. Sci..

[140] Eisen A. (2001). Clinical Electrophysiology of the Upper and Lower Motor Neuron in Amyotrophic Lateral Sclerosis. Semin. Neurol..

[141] Rocha M.C., Pousinha P.A., Correia A.M., Sebastião A.M., Ribeiro J.A. (2013). Early Changes of Neuromuscular Transmission in the SOD1(G93A) Mice Model of ALS Start Long before Motor Symptoms Onset. PLoS ONE.

[142] Wood S.J., Slater C.R. (1997). The contribution of postsynaptic folds to the safety factor for neuromuscular transmission in rat fast- and slow-twitch muscles. J. Physiol..

[143] Andonian M.H., Fahim M.A. (1987). Effects of endurance exercise on the morphology of mouse neuromuscular junctions during ageing. J. Neurocytol..

[144] Tomas J., Batlle J., Fenoll M.R., Santafé M., Lanuza M.A. (1993). Activity-dependent plastic changes in the motor nerve terminals of the adult rat. Biol. Cell.

[145] Tomas J., Santafé M., Lanuza M.A., Fenoll-Brunet M.R. (1997). Physiological activity-dependent ultrastructural plasticity in normal adult rat neuromuscular junctions. Biol. Cell.

[146] Deschenes M.R., Maresh C.M., Crivello J.F., Armstrong L.E., Kraemer W.J., Covault J. (1993). The effects of exercise training of different intensities on neuromuscular junction morphology. J. Neurocytol..

[147] Dorlöchter M., Irintchev A., Brinkers M., Wernig A. (1991). Effects of enhanced activity on synaptic transmission in mouse extensor digitorum longus muscle. J. Physiol..

[148] Husain K., Somani S.M. (1997). Response of cardiac antioxidant system to alcohol and exercise training in the rat. Alcohol.

[149] Miyazaki H., Oh-ishi S., Ookawara T., Kizaki T., Toshinai K., Ha S., Haga S., Ji L.L., Ohno H. (2001). Strenuous endurance training in humans reduces oxidative stress following exhausting exercise. Eur. J. Appl. Physiol..

[150] Holloszy J.O., Oscai L.B., Don I.J., Molé P.A. (1970). Mitochondrial citric acid cycle and related enzymes: Adaptive response to exercise. Biochem. Biophys. Res. Commun..

[151] Acsadi G., Anguelov R.A., Yang H., Toth G., Thomas R., Jani A., Wang Y., Ianakova E., Mohammad S., Lewis R.A. (2002). Increased Survival and Function of SOD1 Mice After Glial Cell-Derived Neurotrophic Factor Gene Therapy. Hum. Gene.

[152] Manabe Y., Nagano I., Gazi M.S.A., Murakami T., Shiote M., Shoji M., Kitagawa H., Setoguchi Y., Abe K. (2002). Adenovirus-mediated gene transfer of glial cell line-derived neurotrophic factor prevents motor neuron loss of transgenic model mice for amyotrophic lateral sclerosis. Apoptosis.

[153] Sun W., Funakoshi H., Nakamura T. (2002). Overexpression of HGF retards disease progression and prolongs life span in a transgenic mouse model of ALS. J. Neurosci..

[154] Drory V.E., Goltsman E., Reznik J.G., Mosek A., Korczyn A.D. (2001). The value of muscle exercise in patients with amyotrophic lateral sclerosis. J. Neurol. Sci..

[155] Pinto A.C., Alves M., Nogueira A., Evangelista T., Carvalho J., Coelho A., de Carvalho M., Sales-Luís M.L. (1999). Can amyotrophic lateral sclerosis patients with respiratory insufficiency exercise?. J. Neurol. Sci..

[156] Gordon T., Tyreman N., Li S., Putman C.T., Hegedus J. (2010). Functional over-load saves motor units in the SOD1-G93A transgenic mouse model of amyotrophic lateral sclerosis. Neurobiol. Dis..

[157] Bello-Haas V.D., Florence J.M., Kloos A.D., Scheirbecker J., Lopate G., Hayes S.M., Pioro E.P., Mitsumoto H. (2007). A randomized controlled trial of resistance exercise in individuals with ALS. Neurology.

[158] Lunetta C., Lizio A., Sansone V.A., Cellotto N.M., Maestri E., Bettinelli M., Gatti V., Melazzini M.G., Meola G., Corbo M. (2016). Strictly monitored exercise programs reduce motor deterioration in ALS: Preliminary results of a randomized controlled trial. J. Neurol..

[159] Meyer R., Spittel S., Steinfurth L., Funke A., Kettemann D., Münch C., Meyer T., Maier A. (2018). Patient-Reported Outcome of Physical Therapy in Amyotrophic Lateral Sclerosis: Observational Online Study. JMIR Rehabil. Assist. Technol..

[160] Merico A., Cavinato M., Gregorio C., Lacatena A., Gioia E., Piccione F., Angelini C. (2018). Effects of combined endurance and resistance training in Amyotrophic Lateral Sclerosis: A pilot, randomized, controlled study. Eur. J. Transl. Myol..

[161] Kaspar B.K., Frost L.M., Christian L., Umapathi P., Gage F.H. (2005). Synergy of insulin-like growth factor-1 and exercise in amyotrophic lateral sclerosis. Ann. Neurol..

[162] Kirkinezos I.G., Hernandez D., Bradley W.G., Moraes C.T. (2003). Regular exercise is beneficial to a mouse model of amyotrophic lateral sclerosis. Ann. Neurol.

[163] Veldink J.H., Bär P.R., Joosten E.A.J., Otten M., Wokke J.H.J., Van Den Berg L.H. (2003). Sexual differences in onset of disease and response to exercise in a transgenic model of ALS. Neuromuscul. Disord..

[164] Mahoney D.J., Rodriguez C., Devries M., Yasuda N., Tarnopolsky M.A. (2004). Effects of high-intensity endurance exercise training in the G93A mouse model of amyotrophic lateral sclerosis. Muscle Nerve.

[165] Carreras I., Yuruker S., Aytan N., Hossain L., Choi J.-K., Jenkins B.G., Kowall N.W., Dedeoglu A. (2010). Moderate exercise delays the motor performance decline in a transgenic model of ALS. Brain Res..

[166] Just-Borràs L., Hurtado E., Cilleros-Mañé V., Biondi O., Charbonnier F., Tomàs M., Garcia N., Tomàs J., Lanuza M.A. (2019). Running and swimming prevent the deregulation of the BDNF/TrkB neurotrophic signalling at the neuromuscular junction in mice with amyotrophic lateral sclerosis. Cell. Mol. Life Sci..

[167] Henriques A., Pitzer C., Schneider A. (2010). Henriques Neurotrophic growth factors for the treatment of amyotrophic lateral sclerosis: Where do we stand?. Front. Neurosci..

[168] Nagy G., Matti U., Nehring R.B., Binz T., Rettig J., Neher E., Sørensen J.B. (2002). Protein kinase C-dependent phosphorylation of synaptosome-associated protein of 25 kDa at Ser187 potentiates vesicle recruitment. J. Neurosci..

[169] Freischmidt A., Wieland T., Richter B., Ruf W., Schaeffer V., Müller K., Marroquin N., Nordin F., Hübers A., Weydt P. (2015). Haploinsufficiency of TBK1 causes familial ALS and fronto-temporal dementia. Nat. Neurosci..

[170] Cirulli E.T., Lasseigne B.N., Petrovski S., Sapp P.C., Dion P.A., Leblond C.S., Couthouis J., Lu Y.-F., Wang Q., Krueger B.J. (2015). Exome sequencing in amyotrophic lateral sclerosis identifies risk genes and pathways. Science.

[171] de Majo M., Topp S.D., Smith B.N., Nishimura A.L., Chen H.-J., Gkazi A.S., Miller J., Wong C.H., Vance C., Baas F. (2018). ALS-associated missense and nonsense TBK1 mutations can both cause loss of kinase function. Neurobiol. Aging.

[172] Pilli M., Arko-Mensah J., Ponpuak M., Roberts E., Master S., Mandell M.A., Dupont N., Ornatowski W., Jiang S., Bradfute S.B. (2012). TBK-1 promotes autophagy-mediated antimicrobial defense by controlling autophagosome maturation. Immunity.

[173] Radtke A.L., Delbridge L.M., Balachandran S., Barber G.N., O’Riordan M.X.D. (2007). TBK1 protects vacuolar integrity during intracellular bacterial infection. PLoS Pathog..

[174] Wild P., Farhan H., McEwan D.G., Wagner S., Rogov V.V., Brady N.R., Richter B., Korac J., Waidmann O., Choudhary C. (2011). Phosphorylation of the autophagy receptor optineurin restricts Salmonella growth. Science.

[175] Korac J., Schaeffer V., Kovacevic I., Clement A.M., Jungblut B., Behl C., Terzic J., Dikic I. (2013). Ubiquitin-independent function of optineurin in autophagic clearance of protein aggregates. J. Cell Sci..

[176] N’Diaye E.-N., Debnath J., Brown E.J. (2009). Ubiquilins accelerate autophagosome maturation and promote cell survival during nutrient starvation. Autophagy.

[177] Rothenberg C., Srinivasan D., Mah L., Kaushik S., Peterhoff C.M., Ugolino J., Fang S., Cuervo A.M., Nixon R.A., Monteiro M.J. (2010). Ubiquilin functions in autophagy and is degraded by chaperone-mediated autophagy. Hum. Mol. Genet..

[178] Wong Y.C., Holzbaur E.L.F. (2014). Optineurin is an autophagy receptor for damaged mitochondria in parkin-mediated mitophagy that is disrupted by an ALS-linked mutation. Proc. Natl. Acad. Sci. USA.

[179] Heo J.-M., Ordureau A., Paulo J.A., Rinehart J., Harper J.W. (2015). The PINK1-PARKIN Mitochondrial Ubiquitylation Pathway Drives a Program of OPTN/NDP52 Recruitment and TBK1 Activation to Promote Mitophagy. Mol. Cell.

[180] Morton S., Hesson L., Peggie M., Cohen P. (2008). Enhanced binding of TBK1 by an optineurin mutant that causes a familial form of primary open angle glaucoma. FEBS Lett..

[181] Ito Y., Ofengeim D., Najafov A., Das S., Saberi S., Li Y., Hitomi J., Zhu H., Chen H., Mayo L. (2016). RIPK1 mediates axonal degeneration by promoting inflammation and necroptosis in ALS. Science.

[182] Řehořová M., Vargová I., Forostyak S., Vacková I., Turnovcová K., Kupcová Skalníková H., Vodička P., Kubinová Š., Syková E., Jendelová P. (2019). A Combination of Intrathecal and Intramuscular Application of Human Mesenchymal Stem Cells Partly Reduces the Activation of Necroptosis in the Spinal Cord of SOD1 G93A Rats. Stem Cells Transl. Med..

[183] Machado C.B., Pluchon P., Harley P., Rigby M., Gonzalez Sabater V., Stevenson D.C., Hynes S., Lowe A., Burrone J., Viasnoff V. (2019). In Vitro Modelling of Nerve-Muscle Connectivity in a Compartmentalised Tissue Culture Device. Adv. Biosyst..

[184] Burden S.J., Yumoto N., Zhang W. (2013). The Role of MuSK in Synapse Formation and Neuromuscular Disease. Cold Spring Harb. Perspect. Biol..

[185] Vilmont V., Cadot B., Vezin E., Le Grand F., Gomes E.R. (2016). Dynein disruption perturbs post-synaptic components and contributes to impaired MuSK clustering at the NMJ: Implication in ALS. Sci. Rep..

[186] Sengupta-Ghosh A., Dominguez S.L., Xie L., Barck K.H., Jiang Z., Earr T., Imperio J., Phu L., Budayeva H.G., Kirkpatrick D.S. (2019). Muscle specific kinase (MuSK) activation preserves neuromuscular junctions in the diaphragm but is not sufficient to provide a functional benefit in the SOD1 G93A mouse model of ALS. Neurobiol. Dis..

[187] Cantor S., Zhang W., Delestrée N., Remédio L., Mentis G.Z., Burden S.J. (2018). Preserving neuromuscular synapses in ALS by stimulating MuSK with a therapeutic agonist antibody. eLife.

[188] Miyoshi S., Tezuka T., Arimura S., Tomono T., Okada T., Yamanashi Y. (2017). DOK7 gene therapy enhances motor activity and life span in ALS model mice. EMBO Mol. Med..

[189] Perez-Garcia M.J., Burden S.J. (2012). Increasing MuSK Activity Delays Denervation and Improves Motor Function in ALS Mice. Cell Rep..

[190] Gorlewicz A., Wlodarczyk J., Wilczek E., Gawlak M., Cabaj A., Majczynski H., Nestorowicz K., Herbik M.A., Grieb P., Slawinska U. (2009). CD44 is expressed in non-myelinating Schwann cells of the adult rat, and may play a role in neurodegeneration-induced glial plasticity at the neuromuscular junction. Neurobiol. Dis..

[191] Takahashi Y., Fukuda Y., Yoshimura J., Toyoda A., Kurppa K., Moritoyo H., Belzil V.V., Dion P.A., Higasa K., Doi K. (2013). ERBB4 mutations that disrupt the neuregulin-ErbB4 pathway cause amyotrophic lateral sclerosis type 19. Am. J. Hum. Genet..

[192] Dols-Icardo O., García-Redondo A., Rojas-García R., Borrego-Hernández D., Illán-Gala I., Muñoz-Blanco J.L., Rábano A., Cervera-Carles L., Juárez-Rufián A., Spataro N. (2018). Analysis of known amyotrophic lateral sclerosis and frontotemporal dementia genes reveals a substantial genetic burden in patients manifesting both diseases not carrying the C9orf72 expansion mutation. J. Neurol. Neurosurg. Psychiatry.

[193] Le Pichon C.E., Dominguez S.L., Solanoy H., Ngu H., Lewin-Koh N., Chen M., Eastham-Anderson J., Watts R., Scearce-Levie K. (2013). EGFR Inhibitor Erlotinib Delays Disease Progression but Does Not Extend Survival in the SOD1 Mouse Model of ALS. PLoS ONE.

[194] Chico L., Modena M., Lo Gerfo A., Ricci G., Caldarazzo Ienco E., Ryskalin L., Fornai F., Siciliano G. (2017). Cross-talk between pathogenic mechanisms in neurodegeneration: The role of oxidative stress in Amyotrophic Lateral Sclerosis. Arch. Ital. Biol..

[195] Boillée S., Cleveland D.W. (2008). Revisiting oxidative damage in ALS: Microglia, Nox, and mutant SOD1. J. Clin. Investig..

[196] Estevez A.O., Morgan K.L., Szewczyk N.J., Gems D., Estevez M. (2014). The neurodegenerative effects of selenium are inhibited by FOXO and PINK1/PTEN regulation of insulin/insulin-like growth factor signaling in Caenorhabditis elegans. Neurotoxicology.

[197] Kim N.C., Tresse E., Kolaitis R.-M., Molliex A., Thomas R.E., Alami N.H., Wang B., Joshi A., Smith R.B., Ritson G.P. (2013). VCP Is Essential for Mitochondrial Quality Control by PINK1/Parkin and this Function Is Impaired by VCP Mutations. Neuron.

[198] Wong Y.C., Holzbaur E.L.F. (2015). Temporal dynamics of PARK2/parkin and OPTN/optineurin recruitment during the mitophagy of damaged mitochondria. Autophagy.

[199] Chouhan A.K., Zhang J., Zinsmaier K.E., Macleod G.T. (2010). Presynaptic Mitochondria in Functionally Different Motor Neurons Exhibit Similar Affinities for Ca2+ But Exert Little Influence as Ca^2+^ Buffers at Nerve Firing Rates In Situ. J. Neurosci..

[200] Recabarren-Leiva D., Alarcón M. (2018). New insights into the gene expression associated to amyotrophic lateral sclerosis. Life Sci..

[201] Léger B., Vergani L., Sorarù G., Hespel P., Derave W., Gobelet C., D’Ascenzio C., Angelini C., Russell A.P. (2006). Human skeletal muscle atrophy in amyotrophic lateral sclerosis reveals a reduction in Akt and an increase in atrogin-1. FASEB J..

[202] Moreno-Igoa M., Calvo A.C., Penas C., Manzano R., Oliván S., Muñoz M.J., Mancuso R., Zaragoza P., Aguilera J., Navarro X. (2010). Fragment C of tetanus toxin, more than a carrier. Novel perspectives in non-viral ALS gene therapy. J. Mol. Med..

[203] Kirby J., Ning K., Ferraiuolo L., Heath P.R., Ismail A., Kuo S.-W., Valori C.F., Cox L., Sharrack B., Wharton S.B. (2011). Phosphatase and tensin homologue/protein kinase B pathway linked to motor neuron survival in human superoxide dismutase 1-related amyotrophic lateral sclerosis. Brain.

